# Spatial distribution and risk assessment of fluorine and cadmium in rice, corn, and wheat grains in most karst regions of Guizhou province, China

**DOI:** 10.3389/fnut.2022.1014147

**Published:** 2022-10-19

**Authors:** Xiangxiang Li, Luoxiong Zhou, Cheng Zhang, Dasuan Li, Zelan Wang, Dali Sun, Chaoxuan Liao, Qinghai Zhang

**Affiliations:** ^1^The Key Laboratory of Environmental Pollution Monitoring and Disease Control, Ministry of Education, School of Public Health, Guizhou Medical University, Guiyang, China; ^2^Guizhou Academy of Testing and Analysis, Guiyang, China

**Keywords:** cadmium, fluorine, grains, spatial distribution, health risk assessment

## Abstract

The pollution status of crops planted in Guizhou province of Southwestern China with high background values of Fluorine (F) and Cadmium (Cd) has attracted people’s concern. The present study aimed to investigate the spatial distributions of F and Cd in rice, corn and wheat grains, and further evaluate their health risks to residents in Guizhou province. The contents of F and Cd were measured by fluoride ion-selective electrode and inductively coupled plasma mass spectrometry (ICP-MS) methods, respectively. Additionally, the inverse distance weighted (IDW) technique was conducted to analyze spatial distribution, and the health risk was estimated by target hazard quotient (THQ) and hazardous index (HI). The results indicate that Cd contents in samples varied from 0.000 to 0.463 for rice, 0.000 to 0.307 for corn, and 0.012 to 0.537 (mg/kg) for wheat, while F contents ranged from 0.825 to 5.193 (rice), 0.946 to 8.485 (corn), and 0.271 to 9.143 (wheat) mg/kg. The Cd exceeding ratios were 11.600% for rice, 13.500% for corn, and 45.100% for wheat grains, respectively. In terms of spatial distribution, high levels of F and Cd in rice were found in the northern and central in Guizhou, while Cd in corn was distributed in the eastern and F in corn were distributed in the west area of Guizhou. Moreover, the high levels of F and Cd in wheat were distributed in the western and eastern areas. The mean carcinogenic risks (R) of Cd in rice, corn, and wheat in children were 4.150 × 10^–4^, 1.670 × 10^–4^ and 3.470 × 10^–4^, respectively, and that in adults were 3.430 × 10^–4^, 0.471 × 10^–4^, and 2.190 × 10^–4^, respectively. The HI for adults in rice, corn and wheat grains were 0.756, 0.154, and 0.514, respectively, and that for children were 0.913, 0.549, and 0.814, respectively. Collectively, the potential risks produced by F and Cd to the local residents should not be ignored.

## Introduction

Fluorine (F) is abundant in the Earth’s crust and considered an essential element for the normal development and growth of humans (1). A suitable dosage of F can prevent dental caries, while excessive absorption can cause dental or skeletal fluorosis in children and adults (2). People can be exposed to F through water, air, food, and other ways. The F background value (929 mg/kg) of soils in Guizhou is higher than that of other areas in China (478 mg/kg) and other nations (20–700 mg/kg) (3). Surveys showed that Guizhou is a high geological background area of F, which may lead to excessive F concentration in plants (4). Furthermore, Guizhou has the phenomenon of coal combustion, which can be hazardous to human health. As a result of this situation, Guizhou has become a region with a high incidence of endemic fluorosis in China.

Cadmium (Cd) is a highly toxic and typical environmental pollutant. It could be accumulated in the human body through the food chain, which is harmful to their health (5). The carbonate rocks in Guizhou province cover 73% of the entire area (6) and the processes of carbonate rock weathering and pedogenesis lead to the enrichment of heavy metals (6). Guizhou is a high geological background area of Cd, and its Cd contents in the environment are higher than in other domestic regions (7). As a result, it may enhance the likelihood of Cd exposure in people. One study showed that the dynamics of exposure to environmental Cd were associated with an increased cancer incidence, and mainly enriched in the kidneys (8). These reasons might lead to a higher prevalence of chronic kidney disease in the carbonate rock areas of the southwest than in other regions in the country.

In addition, a study found that F and Cd frequently coexist in the soils and rhizospheres, and that they have similar relative mobility (9). F and Cd can react to form CdF^+^ complexes under the condition of pH > 6.3 (10), while CdF^+^ was more easily taken up by the plant’s rhizosphere and transferred to crops, potentially causing F and Cd levels in crops to exceed the acceptable limits (11). The previous study showed that concurrent F and Cd might cause stronger toxicity in the liver, kidney function, bones, teeth, and brain (12). The pollution status of crops owing to the accumulation of F had been reported (13). Research showed that rice and wheat pose non-carcinogenic and carcinogenic risks for adults and children due to Cd exposure (14). However, few studies have focused on crop pollution and the impacts of the co-exposure of F and Cd on human health. Therefore, it is critical to evaluate the concurrent levels of F and Cd in crops and the associated health risks to human beings in order to guarantee the health and food safety of residents who rely on rice, corn and wheat grains as staple food.

In view of the discussions above, a total of 334 crop samples (113 for rice, 119 for corn, and 102 for wheat) were collected from Guiyang, Zunyi, Tongren, Anshun, Liupanshui, Qiandongnan, Qianxinan, Qiannan, Bijie, and other regions in Guizhou. The concentrations of F and Cd were determined by fluoride ion selective electrode and inductively coupled plasma mass spectrometry (ICP-MS) methods, respectively. The objectives of this study were to: (i) measure the contents of F and Cd and the single-factor pollution index (Pi) of Cd, (ii) analyze the correlations and spatial distributions of F and Cd, and (iii) assess the non-carcinogenic and carcinogenic risks for adults and children *via* the consumption of these three crops. This study will provide a theoretical basis for maintaining the health of the population in geological background areas with anomalies.

## Materials and methods

### Study area and sample acquisition

**FIGURE 1:**
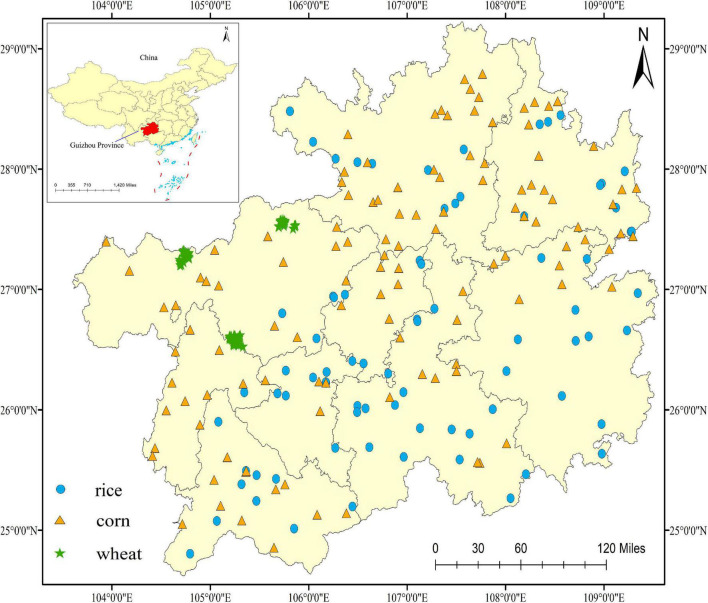
Sampling locations of rice, corn, and wheat grains.

The study area is located in the carbonate rock region of Guizhou province (24°30’–29°13’N, 103°31’–109°30’E), which presents high anomalies of F and Cd. It belongs to a typical karst landform distribution, in which soil types are mainly yellow loam and limestone soils due to the weathering of carbonate rocks. Moreover, the high contents of Ca^2+^ and Mg^2+^ in the soil parent, as well as soil pH values ranging from 7.6 to 8.7, create an environmental condition for the formation of CdF^+^. In addition, the annual average temperature and precipitation are 16°C and 1,449 mm, respectively. The main food crops in investigated area are rice, corn, and wheat grains, and their planting area and annual yield are 6.6 × 10^4^ ha and 4.159 million tons, 5.0 × 10^5^ ha and 2.203 million tons, and 6.6 × 10^5^ ha and 3.337 million tons, respectively. The sampling locations are shown in Figure 1.

In 2021, a total of 334 samples (113 for rice, 119 for corn, and 102 for wheat) were collected using the five-point sampling method at their respective maturity periods, and mixed to form the representative samples (6). It was worth noting that the growth condition of collected samples was normal without apparent damage. Longitude and latitude of each sampling site were recorded by the Global Positioning System (GPS). Then each sample was packaged in a plastic bag and transported to the laboratory. After drying at 72 h for 60°C to hold constant weight, all of them were stored until further analyzed. During the process, all samples were prevented from contacting other materials to avoid pollution.

### Chemical analysis

The F content of the samples was analyzed using the fluoride ion-selective electrode method according to the methods of China (15). A total of 1.00 g of dried sample was added into 10 mL hydrochloric acid (HCl) diluent (HCl: ultrapure water = 1: 11) in a colorimeter tube with a cover and was digested. The sample stood for 1 h and was then metered in a 50 mL volume system by adding ultrapure water.

The Cd content of the samples was determined by ICP-MS (NexION 2000, PerkinElmer, Waltham, USA) according to the multi-element analysis of foods in China (16). A total of 0.20 g of dried sample was digested using 3 mL HNO_3_ in closed poly tetra fluoroethylene and stood for 1 h, and then was ablated for 4 h at 150–170°C. Subsequently, the ablated sample was removed after the poly tetra fluoroethylene cooled down, and the volume was digested with ultrapure water to 10 mL until the sample was measured.

The single-factor pollution index (Pi) is used to evaluate the pollution levels of Cd in rice, corn, and wheat grains. According to the industry standard for monitoring the technical specifications of pollutants in agricultural, livestock and aquatic products (NY/T 398-2000), the Pi was calculated by Eq. (1):


(1) $$\text{Pi}=\frac{\text{Ci}}{\text{Si}}$$

where Pi is the pollution index of the samples (rice, corn, and wheat grains), Ci denotes the average Cd content of rice, corn and wheat grains (mg/kg), and Si represents the Chinese limit levels for Cd (rice for 0.2, corn for 0.1, and wheat for 0.1 mg/kg). The larger the Pi value, the higher the degree of pollution. Pi ≤ 0.7 indicates an excellent quality, 0.7 < Pi ≤ 1 indicates safe, 1 < Pi ≤ 2 indicates slight pollution and Pi > 2 indicates pollution.

### Health risk assessment

The non-carcinogenic risk evaluation was based on the acceptable daily intake (ADI mg/kg/d), target hazard quotient (THQ) and hazardous index (HI) proposed by the Environmental Protection Agency (EPA) in the US (17). Their calculation equations (2–4) are as follows:


(2) $$\text{ADI}=\frac{\text{C}\times \text{IR}\times \text{EF}\times \text{ED}}{\text{BW}\times \text{AT}}$$

where C (mg/kg) denotes the pollutant contents in cereals (corn, wheat, and rice grains) and BW (kg) represents the body weight of adults (59.10 kg) or children (24.90 kg) (18, 19). IR represents the exposure dose, and the average rice, corn and wheat grains consumption of adults and children are 0.389 and 0.198, 0.10, and 0.15, and 0.15 and 0.10 kg/d, respectively (20, 21). EF denotes the exposure frequency (365 d/a), ED denotes the exposure duration of the populace (adults of 70°years and children of 9°years) (18, 19), and AT represents the average exposure duration.


(3) $$\text{THQ}=\frac{\text{ADI}}{\text{RfD}}$$

(4) $$\text{HI}=\sum_{\text{i}=1}^{\text{n}}{\text{THQ}}_{\text{i}}$$

Here, the RfD values of Cd and F are 0.001 and 0.06 mg/kg/d, respectively (22). There may be no health damage to humans when THQ or HI ≤ 1, whereas adverse health damage will occur when THQ or HI > 1.

The carcinogenic risk (R) denotes the potential for a person to develop cancer due to a lifetime of exposure to Cd, and the carcinogenic risk (R) model was used to assess the risk of Cd exposure (23). The R of people was calculated according to Eq. (5):


(5) R=S⁢F×A⁢D⁢I

where SF is the slope factor (mg/kg/d), which is a plausible upper-bound estimate of the probability of a response per unit intake of a chemical over a lifetime (Cd: 0.64) (24). According to the United States Environmental Protection Agency (USEPA, available at http://www.epa.gov), when R < 1 × 10^–6^, the R can be ignored, when R ranges from 1 × 10^–6^ to 1 × 10^–4^, indicating that a cautionary risk is not negligible, and when R > 1 × 10^–4^, indicating that there is an unacceptable risk.

### ArcGIS mapping

The contents of F and Cd in rice, corn, and wheat grains were applied as the input data for spatial distribution analysis. All data were executed by ArcGIS 10.5 (ESRI, Redlands, CA, USA) as previously described methods (25). In the present study, the method of inverse distance weighted (IDW) was used to represent the spatial distribution maps for F and Cd contents in rice, corn, and wheat grains in the study area.

### Statistical analysis

The variance and normality analyses of data were conducted on SPSS 26.0 (SPSS Inc., Chicago, IL, USA). The simple linear regression of GraphPad Prism 9.3.0 was used to calculate the correlation, and Microsoft Office Excel 2019 was used to calculate the mean and median. Nonparametric tests were used to evaluate the significant differences in health risk among different populations. Figures were drawn using GraphPad Prism 9.3.0.

## Results

### Contents of F and Cd in rice, corn, and wheat grains, and the Pi of Cd

As shown in Table 1, the contents of Cd in rice, corn, and wheat grains ranged from 0.000 to 0.463, 0.000 to 0.307, and 0.012 to 0.537 mg/kg, respectively. The F contents in rice, corn and wheat grains varied from 0.825 to 5.193, 0.946 to 8.485, and 0.271 to 9.143 mg/kg, respectively. The contents of F and Cd in crops dropped in the following order: wheat > corn > rice and wheat > rice > corn, respectively. The exceeding percentages of Cd in samples were 11.600% for rice, 13.500% for corn, and 45.100% for wheat grains, according to the food safety standard in China (26). The exceeding percentage of F was not discussed because its food safety standard has been abolished.

**TABLE 1:** The contents of F and Cd in rice, corn, and wheat grains (mg/kg).

Crops	Category^a^	F			Cd			
		Range	Median	Mean	Range	Median	Mean	Exceeding ratio (%)
Rice (*n* = 113)	1	0.825∼1.349	1.054	1.084	0.000∼0.067	0.024	0.028	11.6
	2	1.350∼1.819	1.676	1.643	0.068∼0.129	0.092	0.094	
	3	1.820∼2.087	1.937	1.960	0.130∼0.184	0.149	0.158	
	4	2.088∼2.566	2.292	2.321	0.185∼0.387	0.254	0.269	
	5	2.567∼5.193	2.859	3.065	0.388∼0.463	0.463	0.463	
Corn (*n* = 119)	1	0.946∼1.965	1.783	1.736	0.000∼0.034	0.012	0.014	13.5
	2	1.966∼2.226	2.102	2.095	0.035∼0.069	0.044	0.048	
	3	2.227∼2.593	2.361	2.386	0.070∼0.089	0.085	0.085	
	4	2.594∼3.346	2.987	2.993	0.090∼0.192	0.118	0.127	
	5	3.347∼8.485	5.031	5.219	0.193∼0.307	0.290	0.283	
Wheat (*n* = 103)	1	0.271∼2.547	1.761	1.619	0.012∼0.034	0.022	0.023	45.1
	2	2.548∼3.937	3.083	3.219	0.035∼0.069	0.046	0.050	
	3	3.938∼4.350	4.139	4.158	0.070∼0.099	0.091	0.090	
	4	4.351∼5.353	4.886	4.889	0.100∼0.193	0.140	0.142	
	5	5.354∼9.143	6.376	6.768	0.194∼0.537	0.255	0.290	

^a^The F content range was divided equally into five categories for increased data differentiation because its food safety standard of F has been abolished. For Cd, it is classified into five categories according to the single factor pollution index (Pi) classification: Pi ≤ 0.7 indicates an excellent quality (Dividing them equally into two categories to enhance data differentiation), 0.7 < Pi ≤ 1 indicates safe, 1 < Pi ≤ 2 indicates slight pollution and Pi > 2 indicates pollution.

According to Table 2, the Pi of Cd in rice and corn were 0.408 and 0.434, respectively, indicating they had an excellent quality. On the other hand, the Pi of Cd was 1.352 in wheat, indicating a contamination was observed. These results showed that the pollution of Cd in wheat should be alleviated.

**TABLE 2:** The single factor pollution index of Cd in rice, corn, and wheat grains (mg/kg).

Crops	Number	Cd			Pi	Grade^a^
		Range	Mean	Limit		
Rice	113	0.000∼0.463	0.082	0.200	0.408	excellent quality
Corn	119	0.000∼0.307	0.043	0.100	0.434	excellent quality
Wheat	102	0.012∼0.537	0.135	0.100	1.352	slight pollution

^a^Grade: represents the sample quality.

### Spatial distributions of F and Cd in rice, corn, and wheat grains

**FIGURE 2:**
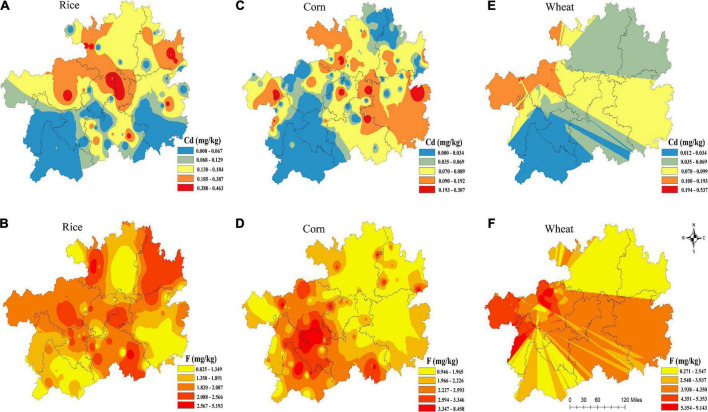
Spatial distributions of F and Cd in rice **(A,B)**, corn **(C,D)**, and wheat **(E,F)** grains.

The spatial distributions of F and Cd contents in rice, corn and wheat grains are shown in Figure 2. Overall, the F and Cd contents in the main edible of rice, corn, and wheat grains in Guizhou province was greater than in other regions of China (27), and the exceeding rate of Cd in crops in western of Guizhou was higher than in other areas.

For rice, the higher Cd values distributed mainly at the northern and central Guizhou (Figure 2A, while the higher *F* values distributed mainly at the west and central Guizhou (Figure 2B. For corn, high Cd levels (Figure 2C were distributed in eastern Guizhou, while its high F levels (Figure 2D) were distributed in western Guizhou. Furthermore, wheat with high Cd and F levels were distributed in west and central Guizhou (Figures 2E,F), respectively.

### Correlations of F and Cd in rice, corn, and wheat grains

**FIGURE 3:**
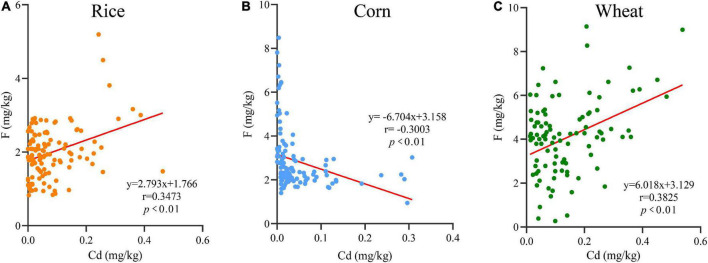
The linear relationships of F and Cd in rice **(A)**, corn **(B)**, and wheat **(C)** grains.

The correlations between Cd and F in rice, corn, and wheat grains are shown in Figure 3. The correlations between F and Cd in grains are shown as positive in rice, negative in corn and positive in wheat, and their regression equations are *y* = 2.793 x + 1.766 (*r* = 0.3473, *p* < 0.01), *y* =−6.704 x + 3.158 (*r* = −0.3003, *p* < 0.01), and *y* = 6.018 x + 3.129 (*r* = 0.3825, *p* < 0.01), respectively. Those results indicated that F and Cd in rice and wheat had a synergistic interaction (*p* < 0.01), which emerged as an antagonistic interaction (*p* < 0.01) in corn.

### Non-carcinogenic and carcinogenic risk of rice, corn, and wheat grains among different populations

**FIGURE 4:**
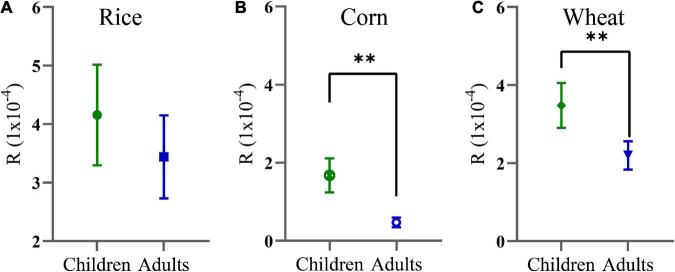
Carcinogenic risks (R) of Cd in rice **(A)**, corn **(B)**, and wheat **(C)** grains. **p < 0.01.

In the present research, only Cd was considered for R. The results showed that the *R* values of Cd in rice, corn, and wheat for children were 4.150 × 10^–4^, 1.670 × 10^–4^, and 3.470 × 10^–4^, respectively, and for adults were 3.430 × 10^–4^, 0.471 × 10^–4^, and 2.190 × 10^–4^, respectively. There was no statistically significant difference in the risk of rice intake between adults and children (Figure 4A). However, the R of children who ingested corn (Figure 4B) and wheat (Figure 4C) grains were greatly higher than that those of adults. The R for children decreased in the order of rice > wheat > corn and decreased in the order of rice > corn and rice > wheat for adults, and there was no statistically significant difference for adults in terms of corn and wheat consumption. The carcinogenic risks of children and adults posed by the consumption of the crops were above their acceptable limits (10^–6^). These results show that a cautionary R existed in the crops, which poses a threat to local citizens, and children were found to be more predisposed than adults.

**FIGURE 5:**
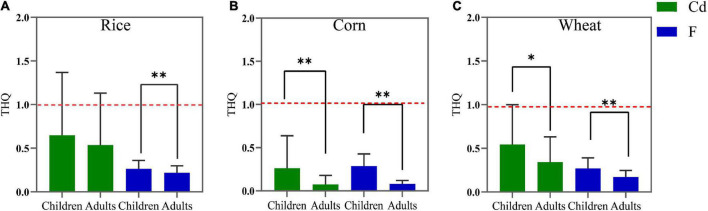
The target hazard quotients (THQ) values for rice **(A)**, corn **(B)**, and wheat **(C)** grains consumption by children and adults. The red dashed line denotes the limit value of the THQ (1.0). *p < 0.05, **p < 0.01.

**FIGURE 6:**
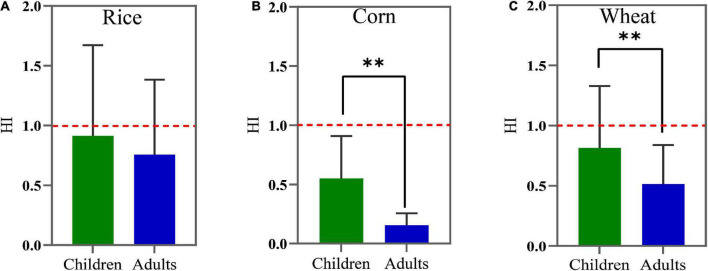
The hazardous index (HI) values for rice **(A)**, corn **(B)**, and wheat **(C)** grains consumption by children and adults. The red dashed line denotes the limit value of HI (1.0). **p < 0.01.

The non-carcinogenic risks of F and Cd in crops estimated by the THQ and HI of children and adults are shown in Figures 5, 6. For children, the mean THQ_*Cd*_ values of rice, corn, and wheat were 0.649, 0.267, and 0.543, respectively, the mean THQ_*F*_ values were 0.264, 0.288, and 0.271, and the mean HI values were 0.913, 0.549, and 0.814, respectively. For adults, the THQ_*Cd*_ values of rice, corn, and wheat were 0.537, 0.074, and 0.343, the mean THQ_*F*_ values were 0.219, 0.081, and 0.171, and the mean HI values were 0.756, 0.154, and 0.514, respectively. The average THQ_*F*_ of crops ingested by adults and children, in descending order, was rice > wheat > corn and corn > wheat > rice, respectively. Additionally, the average THQ_*Cd*_ in adults and children, in descending order, was rice > wheat > corn. The HI of food ingested by adults and children, in descending order, was wheat > rice > corn and rice > wheat > corn, respectively. The average THQ_*F*_ in children was higher than adults when it came to the ingestion of rice (Figure 5A), corn (Figure 5B), and wheat (Figure 5C), and the average THQ_*Cd*_ in children was higher than adults when it came to the ingestion of corn and wheat. There was no statistically significant difference in HI in terms of the intake of rice between adults and children (Figure 6A). However, when children consumed corn (Figure 6B) and wheat (Figure 6C), the HI values were all higher than adults. Therefore, the non-carcinogenic risk assessment confirmed that the adverse effect was observed for local citizens, which suggested that it is critical to minimize the risk of crops in the study area and that more attention should be paid to children.

## Discussion

### Contents of F and Cd in rice, corn, and wheat grains

F and Cd are considered two of the most hazardous environmental pollutants, which has been reported to can induce various health problems. In our results, only the mean Cd level (0.135 mg/kg) in wheat grains was higher than the standard limit of 0.1 mg/kg set by GB 2762-2017, indicating that there might be a health risk posed by Cd in wheat grains. Zhuang et al. (28) study showed that the mean values of Cd in corn were 0.15 mg/kg, which was higher than that in our study. Our findings showed that the average F content was in a downward trend as wheat (4.053 mg/kg) > corn (2.866 mg/kg) > rice (1.993 mg/kg), respectively. Li et al. (22) proved that the F levels in grains were 0.2 mg/kg and greatly lower than our result. Also, research conducted in a West Bengal city showed that the F average contents in *Oryza sativa L* and *Vicia faba* were 0.56 ± 0.14 and 0.71 ± 0.29 mg/kg and appeared the similar results to the former (29), which might be attributed to the high F geological background value of Guizhou.

In addition, the F and Cd levels in the three crops showed different orders. Specifically, the contents of F and Cd declined as the order: wheat > corn > rice and wheat > rice > corn, respectively. Previous studies showed that the Cd absorption of corn was lower than that of vegetables, which was nearly consistent with our research (30, 31). These results might be attributed to the different accumulation abilities for F and Cd existing in different crops. In particular, due to the high geological background values of F and Cd in the Guizhou soils (32), the rhizosphere of crops possibly absorbed high contents of F and Cd from the soils (33), which might make a relatively high accumulation of F and Cd in crops. Furthermore, the average contents of Cd in the edible part of the crops of this study were lower than the soil background values (0.659 mg/kg), which might influence their separate bioavailability (34). It is reported that biochar could significantly reduce the Cd uptake of crops, which might be an important factor to influence bioavailability (35).

### Spatial distributions of F and Cd in rice, corn, and wheat grains

Under the carbonate geology in Guizhou, the distribution of F and Cd contents showed a high trend in the west and a low occurrence in the east (36). However, our findings differentiated from the previous literature. In the case of Guizhou province, yellow soils is widely distributed in the northern, eastern, and central regions, which mainly belongs to acidic soils (37, 38). The evidence proved that the pH of soils was an essential factor that influenced the Cd absorption, which could affect the availability and morphological distribution of Cd in soils (39). Studies confirmed that the effectiveness of Cd increased as the acidity of soils increased, and in turn, the high pH in soils might result in high exchangeable Cd content in soils (33, 40). Bijie city, a wheat-producing area, the geological background of F in the west was higher than that in other regions, and there were gray and yellow clay soils used as cultivated land with larger acidity in the western region (41). Soil acidification increased the bio-efficiency of Cd and promoted its uptake in crops. It may also vary depending on the accumulation site, with the highest F content accumulated in the crop roots, followed by stems, leaves, and edible parts (42). It concluded that what causes the difference in spatial distribution might be put down to the local soil type and acidity. On the other hand, considerable research suggested that phosphate fertilizer is an important factor in regulating the pH of soils. In a previous study, the application of phosphate fertilizer could increase the accumulation of F and Cd in wheat and corn by adjusting the pH in soils (43). Moreover, the study showed that rainfall capacity could be beneficial to the migration and activation of Cd, leading the Cd accumulation in plants (41). Exceptionally, studies showed that some factors including soil parent materials, soil structure, and sand content could affect the bio-effectiveness of F and Cd in soils (44). In summary, it is meaningful and urgent to make clear those factors discussed to comprehensively clarify the accumulation and spatial distribution of F and Cd.

### Correlations between F and Cd in rice, corn, and wheat grains

The correlations between Cd and F in rice and wheat were shown as positive, while in corn were shown as negative. The Cd and F accumulation in oilseed rape had a positive effect described by Li et al., which was consistent with our finding (45). However, Zhang et al study showed that the correlation between F and Cd in corn was negative as the F increased when the Cd decreased (46). These indicated that the interaction of F and Cd in different crops showed different effects. In addition, the physicochemical properties were the essential factors for controlling the variations in F and Cd (47). It is inferred that the specific effects of concurrent F and Cd in crops still exist in some variations. Therefore, it is noteworthy that the simple linear regression does not seem to accurately reflect the relationship enough. In the future study, a pot experiment will be considered to further clarify the correlation between F and Cd. Apart from that, research should be stepped by comprehensively estimating the levels accumulated in various soil types, crop species, and animals, which could provide more persuasive evidence to reveal the correlation between F and Cd.

### Carcinogenic and non-carcinogenic risks of rice, corn, and wheat grains for children and adults

Human health risk estimation is the technique to evaluate the potential adverse health effects in children and adults, which are influenced by environmental chemical components (48). In the present study, the R was conducted to measure the carcinogenic risk. In terms of carcinogenic risk posed by Cd, our results showed that all of the mean *R* values of Cd in the children and adults all exceeded 1 × 10^–4^, indicating that local residents could be suffered from an unacceptable carcinogenic risk in crops. Cd has been listed as a potential carcinogen by USEPA, associated with a series of fatal illnesses such as diseases of bone and the nervous system, and hepatorenal function damage (49, 50). In general, ingestion of Cd-contaminated food at elevated contents might produce a risk to human health. Compared with other regions, previous studies had shown that Cd exposure could trigger cancer risks to the local populace as well (51, 52). Therefore, it is necessary to control the Cd accumulation in the crops and decline its carcinogenic risk to human beings.

Furthermore, THQ and HI are usually applied to assess the non-carcinogenic risk of hazardous materials in foods. Regardless of children or adults, both two values in our results were all lower than 1, indicating there was a low detrimental risk to children and adults due to the F and Cd exposure. Asgari et al. showed that the HI values of Cd were also below 1 (53). And the same reports were found in Liu et al. (54) and Doabi et al. (55). However, the calculated risk in this study was affected by a great degree of uncertainty. Thus, the harmful effects of F and Cd accumulated in the human body for a persisted and long period are not comprehensively considered here.

Totally, the non-carcinogenic and carcinogenic risks of rice, wheat, and corn grains in adults were lower than that in children. The same findings were found in the reports of Ke et al. and Hu et al. (56, 57). This may be related to the children have worse organ tolerance than adults, which are more sensitive to negative factors due to hypoplasia and hypo-immunity (58). In addition, the differences in dietary patterns among the local populace could also lead to differences in human intake. Some studies showed that the dietary pattern of consuming easy-to-roast foods had a significant linear trend with the risk of Fluorosis (59). Finally, some nutrients might reduce the effects of toxic elements. Chen et al. found that a higher dietary intake of specific one-carbon metabolism-related nutrients was associated with a lower prevalence of fluorosis in Guizhou (60). Furthermore, although the average values of THQ_*F*_, THQ_*Cd*_ and HI through oral intake were lower than 1, the risks posed by F and Cd could not be ignored, since human exposure to hazards was not only through ingestion but also enter the body through inhalation and contact, which eventually increase the accumulation of F and Cd in humans. Therefore, it is still limited to evaluate health risks in the present study and more methods should be implemented to comprehensively estimate the risks posed by F and Cd in the future study.

## Conclusion

In the present research, the contents for F and Cd in crops were decreased as wheat > rice > corn and wheat > corn > rice, respectively. The spatial distribution of F and Cd in rice, corn and wheat grains showed different spatial distribution trends. For rice, the high F and Cd contents were distributed in eastern and northern regions, for corn, the high F and Cd contents were distributed in the north and west, for wheat, the high F and Cd levels were distributed in western and eastern regions. Furthermore, the F and Cd contents in rice and wheat were positively correlated and the F and Cd contents in corn were negatively correlated. The R values of Cd in rice, corn, and wheat grains all exceeded acceptable values (10^–6^) for children and adults, posing an unacceptable carcinogenic risk. The results showed that the non-carcinogenic risks estimated by THQ and HI were lower than 1 and the values for children were higher than that higher in adults. Collectively, the persisting health effects will be profound for human beings and thus the corresponding control steps should be implemented to reduce the health risks of F and Cd in Guizhou.

## Data availability statement

The raw data supporting the conclusions of this article will be made available by the authors, without undue reservation.

## Author contributions

QZ: conceptualization, resources, writing—review and editing, supervision, and funding acquisition. XL and LZ: methodology, validation, data curation, and writing—original draft preparation. XL: software and formal analysis. CZ and DL: investigation. CZ and CL: visualization. ZW and DS: project administration. All authors have read and agreed to the published version of the manuscript.
